# Autologous mesenchymal stem cells or meniscal cells: what is the best cell source for regenerative meniscus treatment in an early osteoarthritis situation?

**DOI:** 10.1186/s13287-017-0678-z

**Published:** 2017-10-10

**Authors:** Johannes Zellner, Girish Pattappa, Matthias Koch, Siegmund Lang, Johannes Weber, Christian G. Pfeifer, Michael B. Mueller, Richard Kujat, Michael Nerlich, Peter Angele

**Affiliations:** 1 Experimental Trauma Surgery, Department of Trauma Surgery, University Medical Center Regensburg, Franz Josef Strauss Allee 11, 93042 Regensburg, Germany; 2 Sporthopaedicum Regensburg, Hildegard von Bingen Strasse 1, 93053 Regensburg, Germany

**Keywords:** Meniscus treatment, Tissue engineering, Meniscal cells, Mesenchymal stem cells, Early osteoarthritis, Meniscus regeneration

## Abstract

**Background:**

Treatment of meniscus tears within the avascular region represents a significant challenge, particularly in a situation of early osteoarthritis. Cell-based tissue engineering approaches have shown promising results. However, studies have not found a consensus on the appropriate autologous cell source in a clinical situation, specifically in a challenging degenerative environment. The present study sought to evaluate the appropriate cell source for autologous meniscal repair in a demanding setting of early osteoarthritis.

**Methods:**

A rabbit model was used to test autologous meniscal repair. Bone marrow and medial menisci were harvested 4 weeks prior to surgery. Bone marrow-derived mesenchymal stem cells (MSCs) and meniscal cells were isolated, expanded, and seeded onto collagen-hyaluronan scaffolds before implantation. A punch defect model was performed on the lateral meniscus and then a cell-seeded scaffold was press-fit into the defect. Following 6 or 12 weeks, gross joint morphology and OARSI grade were assessed, and menisci were harvested for macroscopic, histological, and immunohistochemical evaluation using a validated meniscus scoring system. In conjunction, human meniscal cells isolated from non-repairable bucket handle tears and human MSCs were expanded and, using the pellet culture model, assessed for their meniscus-like potential in a translational setting through collagen type I and II immunostaining, collagen type II enzyme-linked immunosorbent assay (ELISA), and gene expression analysis.

**Results:**

After resections of the medial menisci, all knees showed early osteoarthritic changes (average OARSI grade 3.1). However, successful repair of meniscus punch defects was performed using either meniscal cells or MSCs. Gross joint assessment demonstrated donor site morbidity for meniscal cell treatment. Furthermore, human MSCs had significantly increased collagen type II gene expression and production compared to meniscal cells (*p* < 0.05).

**Conclusions:**

The regenerative potential of the meniscus by an autologous cell-based tissue engineering approach was shown even in a challenging setting of early osteoarthritis. Autologous MSCs and meniscal cells were found to have improved meniscal healing in an animal model, thus demonstrating their feasibility in a clinical setting. However, donor site morbidity, reduced availability, and reduced chondrogenic differentiation of human meniscal cells from debris of meniscal tears favors autologous MSCs for clinical use for cell-based meniscus regeneration.

## Background

The meniscus is a tissue located between the femoral condyle and tibial plateau of the knee and aids in the force transmission, shock absorption, joint stability, lubrication, and proprioception of the knee joint [1]. The meniscus is composed of two compartments: an inner avascular region and a vascularized outer zone. The composition of these regions varies, whereby the inner meniscus resembles articular cartilage with predominantly collagen type II and proteoglycans, whilst the outer meniscus is similar to fibrocartilage with a high proportion of collagen type I [2]. Meniscal tears are common within the knee joint, particularly in sports and high-impact trauma. The localization of the injury determines the healing capacity of the meniscus. Lesions in the vascularized zone have the ability to be successfully repaired via suturing. However, tears within the avascular region have limited capacity for repair due to poor intrinsic repair capacity [3]. Loss of meniscal substance within this region overloads the underlying articular cartilage [4], increasing the chances for early onset osteoarthritis [5]. Methods for the treatment of meniscus in a clinical situation have been described, including tissue engineering approaches [6, 7]. However, there is no consensus on the appropriate cell type to be utilized in an autologous situation.

In a clinical situation, meniscal tears have been correlated with the onset of early osteoarthritis [8, 9]. Due to delayed diagnosis, many meniscal lesions have to be treated in an environment with a disturbed joint homeostasis caused by early degenerative changes. In such a situation additional removal of meniscus tissue would only exacerbate the cartilage osteoarthritic state and lead to the collapse of the knee joint [10]. Although regenerative treatment of the meniscus in such an early degenerative environment is demanding, the aim of restoration of as much meniscus tissue as possible has to be proposed. Tissue engineered solutions have the potential to naturally heal the defect and also to protect the surrounding cartilage tissue from further damage.

Tissue engineering approaches represent a novel means for the regeneration of meniscal defects. These may be placed into two categories: cell-based and cell therapeutic approaches, with the former being the most promising approach at present. Regenerative meniscus treatment strategies provide a promising option, particularly for treatment of critical defects in the avascular region or even an early osteoarthritic situation. Recent preclinical studies have shown promising results regarding the regeneration of meniscal defects in the vascular region but also in the avascular part using a previously described approach [11–14].

However, as different cell sources have been used to enhance meniscal regeneration within these experimental settings, it needs to be ascertained which cells provide the best treatment for meniscus defects in the context of clinical application. Meniscal cells may be isolated from the tissue and then reinserted within a carrier back into the patient. These cells have been evaluated in vitro, whilst also demonstrating multilineage differentiation potential [15–17]. However, there is a lack of in-vivo data concerning the use of meniscal cells for tissue repair. Many translational studies have focused on the use of mesenchymal stem cells (MSCs) due to their ability to differentiate towards multiple lineages following extensive in-vitro expansion. They have also been shown to have a high chondrogenic potential under in-vitro conditions whilst having the capacity to have a meniscus-like phenotype [18]. Gonzalez-Fernandez et al. evaluated bone marrow- or adipose-derived MSCs seeded into a collagen scaffold and then inserted into a meniscus defect created within a horse model [19]. Results demonstrated that there was no difference in tissue regeneration 12 months postoperatively, agreeing with results from a previous investigation [20]. MSCs have also been derived from the synovial fluid and have been shown to increase following meniscal tears [21]. Furthermore, investigators have used MSCs derived from the synovial fluid and shown good outcomes in animal models 12 months postoperatively [22, 23].

The present study sought to evaluate, in a clinical situation, the feasibility of autologous cell-based tissue engineering strategies for treating an avascular meniscus defect in a knee with early degenerative changes. With this aim, an early osteoarthritis situation was created by resection of both medial menisci. A punch defect was inserted in the avascular region of a rabbit lateral meniscus. Bone marrow and meniscus tissue were harvested from the operated rabbit, and the cells were expanded and reimplanted in the joint for assessment. In conjunction, human meniscal cells from nonrepairable bucket tears and bone marrow cells were assessed with respect to their meniscal potential under in-vitro conditions.

It was hypothesized that meniscus regeneration is also possible in the environment of early osteoarthritis of the knee. Both cell types, meniscal cells and MSCs, should be capable of promoting meniscal healing in an animal model within this demanding situation. However, donor site morbidity and reduced potential of human meniscal cells for chondrogenic differentiation might limit their use at the injury site and favor a stem cell-based treatment approach for meniscal defects in a clinical setting.

## Methods

### In-vivo model

#### Cell harvest and culture—animal trial

Bone marrow-derived autologous MSCs were harvested approximately 4 weeks prior to meniscus defect treatment. The bone marrow harvest and cell isolation were performed as previously described [24]. In brief, rabbits were anesthetized intramuscularly using a combination of 0.6 ml/kg ketamine 10% and xylazin 2%. The bone marrow was harvested from the iliac crest via a small incision into the bone cortex with an 18G needle and collected into a syringe containing heparin. Culture media was added to the aspirate and 20 × 10^6^ nucleated cells were plated into culture flasks and cultivated at 37 °C. Dulbecco’s modified Eagle’s medium (DMEM) low glucose concentration supplemented with 10% fetal bovine serum (FBS), 1% penicillin, and 1% HEPES was added to the aspirate. Following first refreshment after 7 days, adherent cells were described as MSCs.

Meniscus cells were harvested from the complete medial meniscus of both knees that were resected via arthrotomy during the same surgery as the bone marrow harvest. The menisci were minced and digested in collagenase solution overnight. Following centrifugation at 1000 rpm for 10 min, the cells were resuspended and cultured with serum-supplemented RPMI-1640 (10% FBS, 100 U/ml penicillin, 100 ug/ml streptomycin, 0.292 mg/ml l-glutamine, 2.383 mg/ml HEPES) at 37 °C and 5% CO_2_. Media changes for both cell types were carried out twice a week. Cell harvest was performed when the cultured cells had reached 80% confluence.

#### Surgical procedure for meniscus punch defects

Rabbit animal models have been described and are validated models for testing of menisci treatment in the avascular zone [11–13]. Similar to untreated human menisci, untreated defects in the avascular zone or filling with a cell-free implant showed no tendency for healing [11]. The procedures were approved by the Institutional Animal Care and Use Committee at our institution.

Twelve New Zealand White rabbits (5-month-old males) were used in this study. The rabbits were anesthetized and exposure of the lateral joint compartment was achieved by a lateral parapatellar arthrotomy. Using limited soft tissue release, the lateral meniscus was luxated anteriorly and avascular meniscal defects made by using a 2-mm punch device (Stiefel, Offenbach am Main, Germany).

On the one side, the punch defect was treated by an autologous MSC matrix composite and, on the contralateral knee, the punch defect in the lateral meniscus was filled with an autologous meniscal cell matrix composite. Fixation was achieved by press-fit implantation of the cell-matrix constructs. Following relaxation of the meniscus, the joint capsule was reattached and skin closure was achieved. Postoperatively the animals were allowed free movement without use of any immobilization.

The animals were sacrificed at 6 or 12 weeks; each group consisted of 6 New Zealand White Rabbits.

#### Composite scaffolds/cell carrier and cell seeding

Sponge scaffolds were formulated as a cell carrier for the study and manufactured from 70% derivatized hyaluronan-ester and 30% gelatin, as previously described [25, 26]. The hyaluronan component was obtained from the commercially available product, Jaloskin (Fidia Advanced Biopolymers, Abano Terme, Italy), which is manufactured from sodium hyaluronate and highly esterified with benzyl alcohol on the free carboxyl groups of glucoronic acid within the polymer. The gelatin component was hydrolyzed bovine collagen (Sigma, Taufkirchen, Germany). Porous scaffolds were manufactured by solvent casting via a particulate leaching technique, using NaCl with controlled grain size as the porogen. The primary pore size was 250–350 μm and secondary pore size was 50–100 μm. Scaffolds had a diameter of 2.2 mm and a height of 3 mm.

#### Cell loading of composite scaffolds

MSCs and meniscal cells were loaded onto the scaffolds as previously described [13, 25]. Briefly, MSCs and meniscal cells were trypsinized, counted, washed, and resuspended in DMEM-high glucose at a concentration of 1.0 × 10^6^ cells into the composite scaffolds. The cell-scaffold constructs were implanted in the meniscus punch defects without preculture [11].

#### Gross assessment of joint morphology

Rabbits with surgical implants were euthanized for tissue harvest with an overdose of pentobarbital (1600 mg/ml) given intraperitoneally. Following exposure of the knee joint, the macroscopic morphology of the meniscus and the attachments of the meniscus to the tibial plateau and the femoral condyles were evaluated and photographed.

#### Histology

The lateral menisci harvested from the in-vivo experiments were fixed in 4% phosphate-buffered paraformaldehyde, embedded in Tissue-Tek OCT and frozen in liquid nitrogen. Radial sections (10 μm) of all samples were produced and stained with toluidine blue.

All distal femurs harvested from the in-vivo experiments were prepared according to the OARSI histological cartilage pathology assessment protocol [27]. Samples were fixed for 24 h in 4% phosphate-buffered saline (PBS)-buffered paraformaldehyde and then decalcified in 10% equivalent ethylenediaminetetraacetic acid (Sigma, Taufkirchen, Germany) at pH 8. After decalcification the femoral condyles were embedded, cut, and stained with Safranin O (Sigma, Taufkirchen, Germany). The grade of osteoarthritic change of all femoral condyles was analyzed by the established OARSI grading for osteoarthritis cartilage histopathology [27].

#### Immunohistochemistry

As the pars intermedia of the rabbit meniscus contains mainly collagen type II, specifically towards the avascular central part of the meniscus, the immunohistochemical analysis was performed for collagen type II. Sections were washed and then digested for 15 min with 0.1% pepsin at pH 3.5 to facilitate antibody access to the target epitopes. Type II collagen was immunolocalized by the immunoperoxidase ABC technique (Vector, Burlingame, CA, USA), applying monoclonal primary antibodies ms. anti-collagen II, clone II-4C11 (Calbiochem, Merck, Schwalbach, Germany), biotin-conjugated polyclonal secondary antibodies (goat anti-mouse IgG; Jackson, West Grove, PA, USA), and the nickel- and cobalt-enhanced DAB stain visualization.

#### Meniscus scoring system

In order to compare the macroscopical, histological, and immunohistochemical results after repair of meniscal lesions, a validated meniscus scoring system was used that was developed and published for the evaluation of meniscal defects [11, 12, 28]. Subgroups in macroscopical assessment were “stability” and “defect filling with repair tissue”; for histological analysis the “quality of the surface area”, “integration”, “cellularity”, “cell morphology”; and for immunohistochemical characterization the “expression of proteoglycan and moderate collagen type II in the repair tissue”. The repair was graded by summing up the scores from 0–3 of eight individual subgroups. Consequently, the final scores were between 0 points (no repair) and maximal 24 points (complete reconstitution of the meniscus). The data were collected from two blinded scorers, both experienced in knee anatomy of rabbits and in histological assessment.

### In-vitro model

#### Cell harvest and aggregate preparation—human cells

The procedures were approved by the local Ethics Committee Review Board. Human meniscal specimens were obtained, after written consent, from 14 patients (average age 36.6 years) undergoing arthroscopy of the knee with the approval of the local Ethics Committee. If a bucket handle tear of a meniscus was considered as nonrepairable, these meniscal parts were harvested for further in-vitro analysis. Meniscal cells were isolated as described in the in-vivo animal study. In all cases, meniscal cells between passages 1 and 3 were used for in-vitro assessments.

Human MSCs were extracted, after written consent, from bone marrow samples of six patients (average age 32 years) obtained through an iliac crest puncture prior to bone graft harvest for back surgery. Initially, MSCs were isolated from bone marrow aspirate by Ficoll density-gradient centrifugation. Cells were seeded in 75-cm^2^ tissue culture flasks and maintained at 37 °C in a humidified atmosphere containing 5% CO_2_. Expansion medium consisted of DMEM (Gibco Invitrogen, Karlsruhe, Germany) supplemented with 10% FBS (PAN Biotech, Aidenbach, Germany) and 10% penicillin/streptomycin (Gibco Invitrogen). During expansion the medium was replaced twice a week.

Cell harvest was performed when the cultured meniscal cells or MSCs had reached 80% confluence. Aggregates of the different cell types (2 × 10^5^ cells/500 μl medium) were centrifuged at 1000 rpm for 10 min and then cultured in chondrogenic medium with serum-free high-glucose DMEM (Gibco, Invitrogen) containing 100 nM dexamethasone (Sigma, Steinheim, Germany), 1% ITS + 3 (insulin–transferrin–selenium solution; Sigma), 200 mM l-ascorbic acid 2-phosphate (Sigma), 1 mM sodium pyruvate (Gibco Invitrogen), and 10 ng/ml human transforming growth factor (TGF) beta-1 (R&D Systems, Wiesbaden, Germany) for 21 days. A total of 16 aggregates of each cell source from each patient were analyzed at days 0, 7, 14, and 21.

#### Macroscopic evaluation and histology–human cells

Aggregates were fixed in 4% PBS-buffered paraformaldehyde and then infiltrated with increasing concentrations (10–30%) of sucrose. Following photographic documentation, aggregates were embedded in Tissue-Tek (Sakura, Zoeterwoude, The Netherlands) and cryosectioned at 10–12 μm with an HM 500 OM cryotome (Microm, Berlin, Germany). The metachromatic dye 1,9-dimethylmethylene blue (DMMB; Sigma) was used to detect and analyze the synthesized sulfated glycosaminoglycans (sGAG).

#### Immunohistochemistry (human collagen type I and II)

Sections were stained with monoclonal antibody against type I and II collagen (mouse anti-type I collagen (1 ug/ml, Calbiochem) or mouse anti-type II collagen IgG1 (1:100; Calbiochem, Darmstadt, Germany)) after predigestion with 0.1% pepsin (Sigma) in 1× citric/phosphate McIlvaine buffer (pH 3.6) for 15 min. Incubation with primary antibody was carried out overnight at 4 °C. Biotinylated secondary antibody (goat anti-mouse IgG at a dilution of 1:100) was detected with a horseradish peroxidase (HRP)-labeled avidin–biotin complex and diaminobenzidine tetrahydrochloride hydrate substrate.

#### Biochemical analyses

Aggregates were homogenized in 0.05 M acetic acid plus 0.5 M NaCl, digested with 10 mg/ml pepsin, and dissolved in 0.05 M acetic acid on a rotator for 48 h at 4 °C. The next steps of digestion were performed as described in the Native Type II Collagen Detection Kit 6009 protocol (Chondrex, Redmond, WA, USA).

The sGAG content of the digests was measured using a colorimetric assay with DMMB. The amount of synthesized type II collagen of the aggregates was determined with the Native Type II Collagen Detection Kit. In addition, the digests were assayed for DNA concentration using the Quant-iT dsDNA Assay Kit (Invitrogen, Eugene, OR, USA).

#### RNA isolation, cDNA synthesis, and gene expression analysis

Eight to ten aggregates per condition and time point (day 0 and day 21) for each donor were pooled and homogenized using a precooled Precellys homogenizer with Precellys Ceramic Kit 1.4/2.8 mm. RNA was isolated with the RNeasy Plus Universal Mini Kit (Qiagen) according to the manufacturer’s instructions. Reverse transcription was performed with the Transcriptor First-Strand cDNA Synthesis kit (Roche). Semiquantitative real-time polymerase chain reaction (PCR) was performed with Brilliant SYBR Green QPCR mix (Stratagene) on the Biorad CFX96 System. Gene expression was normalized to three different reference genes (vacuolar protein sorting 29 homolog (VPS29), proteasome subunit beta type 4 (PSMB4), and receptor accessory protein 5 (REEP5)) using the delta-delta-Ct method. Primer sequences were: PSMB4, forward gcttagcactggctgcttct, reverse cgacatgcttggtgtagcct; VPS29, forward agctggcaaactgttgcac, reverse gacggtggtggtgactgag; REEP5, forward aggtcagccactgggtatca, reverse cctctctcctctgcaacctg; col2, forward gggcaatagcaggttcacgta, reverse tgtttcgtgcagccatcct

### Statistical analysis

In-vitro human data were normalized and compared using the two-tailed Mann-Whitney *U* test (SPSS 15.0 Software; SPSS, Chicago, IL, USA). In-vivo test scoring results for the stem cell-treated groups and meniscal cell-treated groups were compared by paired *t* tests. All evaluations and levels of statistical significance were set at a probability value of less than 0.05.

## Results

### Gross assessment of rabbit knee joints

**Fig. 1:**
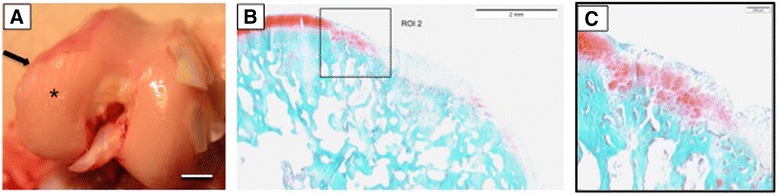
**a** Macroscopic view of femoral condyles 3 months after harvesting the medial meniscus showing early osteoarthritic changes: cartilage degeneration (*asterisk*) and osteophyte formation (*arrow*) on the medial side are detectable. *Scale bar* = 5 mm. **b** Histological image of the degenerated area of the femoral condyle showing early osteoarthritis changes. *Scale bar* = 2 mm. **c** Under higher magnification an OARSI grade 3 cartilage pathology with fissures extending into the deep zone can be observed. *Scale bar* = 0.2 mm. The average OARSI grading of all 12 knees at 3 months was 3.1

To harvest a sufficient number of meniscal cells for the cell-based treatment the total resection of both medial menisci was necessary. Macroscopically, the gross assessment of the rabbit knee joints revealed increasing degenerative changes in all cases over time. Essentially, after 3 months the medial compartments of the knees showed early osteoarthritic changes with cartilage abrasion, chondral defects, and softening of the surrounding cartilage. Small osteophytes were detected mainly in the medial compartment (Fig. 1) as signs of early degenerative changes.

Using the histological OARSI grading system all femoral condyles showed moderate osteoarthritic signs with Safranin O staining, with discontinuity or erosion of the cartilage surface and vertical fissures extending to the mid- or deep zone (Fig. 1). The average grading was 3.1, indicating an early osteoarthritis situation.

### In-vivo repair of meniscus punch defects by meniscal cell- or MSC-based treatment

**Fig. 2:**
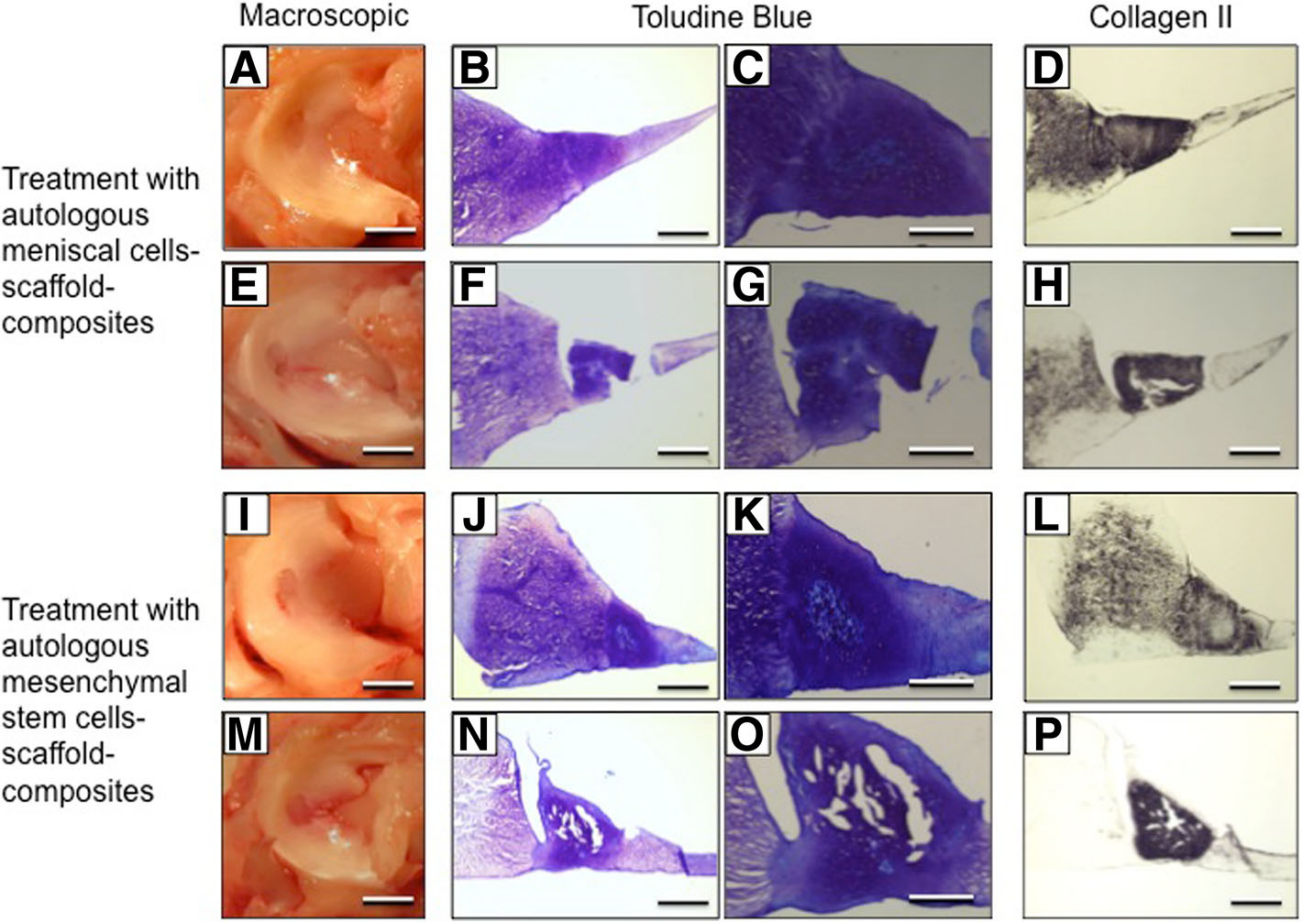
Macroscopic, histological, and immunohistochemical treatment results of 2-mm circular meniscus defects in the avascular zone with meniscus cell-scaffold composites (**a**–**h**) and MSC-scaffold composites (**i**–**p**). In both groups (each *n* = 6), successful meniscus regeneration with differentiated repair tissue could be detected after 3 months in vivo. Most of the treated menisci show promising treatment results (images **a**–**d**, best results after meniscus cell treatment; **i**–**l**, best results after MSC treatment) with completely integrated meniscus-like regenerated tissue (**e**–**h** and **m**–**p** show the worst results of each group). *Scale bars*: **a**,**e**,**i**,**m** = 4 mm, **b**,**d**,**f**,**h**,**j**,**l**,**n**,**p** = 0.5 mm, **c,g,k,o** = 0.1 mm). **a**,**e**,**i**,**m** Macroscopic view of menisci on the tibia plateau with filled circular defects after 3 months; **c,g,k,o** Higher magnification of the integration zones of images **b,f,j,n. d,h,l,p** Collagen type II immunostaining

Six weeks after treatment of a meniscus punch defect by implantation of a hyaluronan collagen composite matrix seeded with autologous meniscal cells, the defects were partially filled with undifferentiated tissue. Repair tissue showed a lack of integration mainly towards the tip of the meniscus. Three months after treatment, the meniscus punch defect in the avascular zone was completely filled with repair tissue. Histologically, the defect was filled with differentiated meniscus-like tissue. The de novo repair tissue was totally integrated with the surrounding native meniscus both at the base and also at the tip of the meniscus. Immunohistochemistry also revealed differentiation of the repair tissue with positive staining for collagen II (Fig. 2a–h).

In comparison, meniscal punch defects treated with MSCs showed partial defect filling with incomplete tissue differentiation of the repair tissue after 6 weeks following repair. After 3 months of treatment, meniscal defects were completely filled with dense repair tissue with stable integration to the native meniscus. Histologically, the regenerated tissue was meniscus-like with a low cell density but typical pericellular meniscal cavities and an extensive amount of extracellular matrix. Immunostaining for collagen II was moderately positive which is typical for rabbit menisci in the pars intermedia. The reconstitution of meniscus architecture with typically radially orientated collagen fibers could be observed (Fig. 2i–p).

### Scoring results of the meniscal repair tissue

**Fig. 3:**
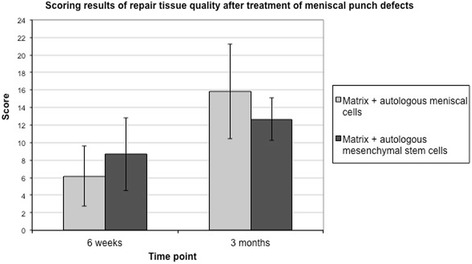
Scoring results of the repair tissue quality after 6 weeks (*n* = 6 rabbits) and 3 months (*n* = 6 rabbits) in vivo. No statistical difference was observed between the meniscal cell- and the MSC-treatment groups

Meniscal tissue regeneration induced by the different cell-based repair strategies were compared and analyzed by a validated and published meniscus scoring system. The scoring results for both groups were high, particularly after 3 months, indicating good meniscus regeneration with differentiated tissue. Scoring values between 12 and 16 points were observed. No statistically significant difference between the meniscal cell- and MSC-based treatments could be detected after 6 weeks (*P* > 0.005) or 3 months (*P* > 0.005) (Fig. 3).

Previous studies showed a reduced repair capacity for untreated avascular meniscal defects or defects treated by a cell-free scaffold in the same rabbit model. The induced repair tissues in these situations demonstrated poor quality with undifferentiated and nonintegrated fibrotic defect filling. The average score of the regenerated defects in these historical controls remained at 8 points after 6 weeks and showed no further changes after 3 months [11]. Hence, a control with untreated or cell-free scaffolds was not used in this study.

### MSCs demonstrate greater chondrogenic potential compared to meniscal cells

**Fig. 4:**
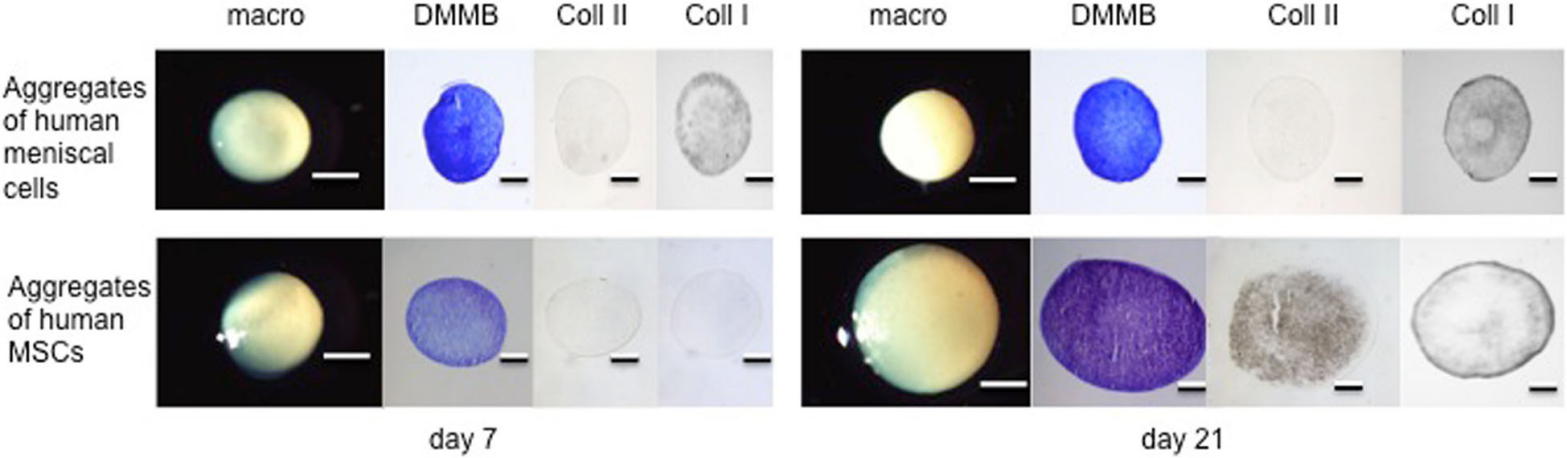
Representative macroscopic and histological images of cell aggregates of human meniscal cells (three pellets of each donor (*n* = 14)) from non-refixable meniscal tears (*upper row*) and human mesenchymal stem cells (*MSCs*; three pellets of each donor (*n* = 6); *lower row*) at day 7 and day 21. The meniscal cells show no increase in size and no chondrogenic differentiation from day 7 to day 21. No positive staining for collagen II (*Coll II*) can be detected. Human MSC aggregates show an increase in size and chondrogenic differentiation during the culture period. At day 21 a positive staining for collagen II of the whole aggregate can be observed. Collagen I (*Col I*) staining was positive at the surface of the MSC pellets. *Scale bars*: macroscopic images = 1 mm, histological images = 0.5 mm. *DMMB* 1,9-dimethylmethylene blue

**Fig. 5:**
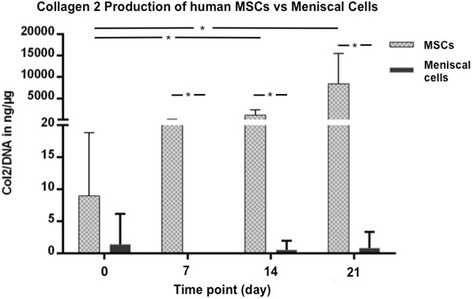
Comparison of collagen II (*Col2*) ELISA results of aggregates from human meniscal cells (*black*) (three pellets at each time point of each patient (*n* = 14)) and mesenchymal stem cells (*MSCs*; *gray*) (three pellets at each time point of each patient (*n* = 6)) after 21 days of culture in chondrogenic media. Meniscal cells from non-refixable meniscal tears show no chondrogenic potential. *P < 0.005

**Fig. 6:**
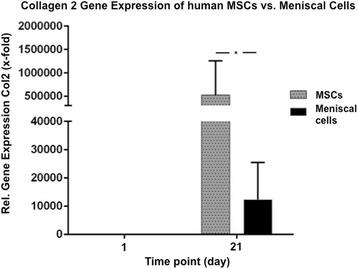
Comparison of real-time PCR analysis for collagen type II (*Col2*) expression of aggregates from human meniscal cells (*black*) (10 pellets at each time point of each donor (*n* = 14)) and mesenchymal stem cells (*MSCs*; *gray*) (eight pellets at each time point of each donor (*n* = 6)) after 21 days of culture in chondrogenic media. At day 21, MSCs showed a significantly higher relative gene expression and collagen type II upregulation compared to meniscal cells derived from non-refixable meniscal tears. *P < 0.005

Passages 1–3 were required to achieve the necessary number of cells from the arthroscopically resected native meniscus tissue to create meniscal cell aggregates. Macroscopically and histologically, no increase in size or chondrogenic differentiation via DMMB and collagen II staining could be detected over 21 days (Fig. 4), whilst collagen I was detected within the matrix. Enzyme-linked immunosorbent assay (ELISA) testing at day 0 revealed only a low amount of collagen II, and no increase in collagen II normalized to DNA could be seen at days 7, 14, or 21 compared to day 0 (Fig. 5). Real-time PCR revealed a moderate upregulation of collagen Type II expression at day 21 (Fig. 6).

In comparison to meniscal cell aggregates, human MSCs showed a macroscopic increase in size and chondrogenic differentiation in the DMMB or collagen type II staining over 21 days (Fig. 4). Collagen type I could only be detected at the surface of the MSC pellets after 21 days.

ELISA testing detected significantly (*P* < 0.005) higher amounts of collagen II in MSC aggregates compared to the meniscal cell pellets at all time points (days 0, 7, 14, and 21) of culture and a fast and high increase in collagen II content at days 7, 14, and 21 compared to day 0 (Fig. 5). In real-time PCR analysis, MSCs showed a significantly (*P* < 0.005) higher upregulation of collagen type II expression at day 21 compared to the meniscal cell aggregates (Fig. 6).

These results indicate a significantly higher chondrogenic potential of human MSCs compared to human meniscal cells.

## Discussion

Tissue engineering approaches for the treatment of meniscal defects have demonstrated a promising means of restoring native meniscus properties, especially injuries to the inner avascular region. In particular, treatment of meniscus injuries within the inner avascular region could utilize this technique. However, to evaluate translational approaches, models mimicking the degenerative situation are required. The present investigation sought to evaluate cell-based tissue engineering approaches for treatment of the avascular region of the meniscus within a rabbit model showing signs of early osteoarthritis. The present study demonstrated regenerative treatment of avascular meniscal defects in this situation for two different autologous cell sources, meniscal cells and MSCs that were seeded in a hyaluronan collagen composite matrix. Three months of in-vivo treatment with either cell type enabled defect filling with differentiated meniscus-like tissue that was completely integrated with the surrounding native tissue.

Early osteoarthritic changes of the knee are a very demanding situation, especially for regenerative treatment strategies [8]. Many of these degenerative changes might initiate an inflammatory status and secretion of catabolic factors that lead to development of late-stage osteoarthritis [10]. In the context of early osteoarthritis, significant correlations between early osteoarthritic changes in the submeniscal tibial plateau cartilage and meniscal degeneration have recently been detected [29]. Additionally, there appears to be a correlation between meniscal extrusion and cartilage damage in the peripheral region of the tibial plateau, underlining the fact that the submeniscal region is vulnerable to early osteoarthritis [30], and thus leads to structural and mechanical alterations of the meniscus leading to further implications within the joint [31, 32]. Although the onset of joint degeneration represents a very demanding situation for regenerative treatment, these facts emphasize the need to restore the meniscus to prevent knee collapse. Resections of both medial menisci in the animal model in this study leads to early degenerative changes in all rabbit knees after 3 months, with cartilage defects and formation of osteophytes. The average OARSI grading was 3.1, indicating an early OA situation [27] (Fig. 1).

Meniscal cells are derived from the tissue at the defect site and are a potential cell source for treatment of meniscal injuries, although the meniscal self-healing capacity is limited [1, 33]. Using a tissue engineering approach helps to overcome these limitations, with a reduced requirement for intrinsic meniscal regeneration. The application of autologous meniscal cells seeded on a hyaluronan collagen-based scaffold induced complete defect filling with differentiated tissue. Webber [34] showed that culture and differentiation of human meniscal cells was possible. However, monolayer expansion of the cells has been shown to result in dedifferentiation and thus requires three-dimensional culture to restore phenotype [35]. Furthermore, regional differences regarding the chondrogenic potential exist, as meniscal cells derived from the outer vascularized zone show a higher chondrogenic capacity than meniscal cells from the inner avascular part [16]. Thus, meniscal cells alone are not wholly responsible for the reduced intrinsic self-healing capacity. Hennerbichler et al. [15] showed that reinserted meniscal plugs in the outer and inner zones of the meniscus reintegrated into the surrounding meniscal tissue in vitro, with stable connecting fibers between the meniscal cells. Explants from the avascular inner zone and vascular outer zone of the meniscus exhibit similar healing potential and repair strength in vitro. In the present investigation, we have shown the repair capacity of the meniscus cells in an in-vivo situation. The reason for the enhanced repair in these previous investigations may be the existence of progenitor populations within the meniscus, particularly from the outer meniscus [1, 17].

A substantial disadvantage of autologous meniscal cells as a source for cell-based treatment is their limited availability and the resultant donor-side morbidity. In the in-vivo study, the resection of the medial menisci of both knee joints was necessary to obtain a sufficient number of cells for a cell-based treatment of a small 2-mm circular punch defect. Three months postoperatively, all knee joints began to show degenerative changes in the medial compartment with chondral lesions, softening of the surrounding cartilage, and formation of osteophytes. This is not surprising since the resection of the medial meniscus serves as a model for inducing the development of osteoarthritis in animal studies [36]. In clinical practice, the only possible option to obtain autologous meniscal cells would be to harvest meniscal debris or tissue derived from nonrepairable tears. Baker et al. described that cells derived from surgical debris are a potent cell source for engineered meniscus constructs in vitro [37]. However, their results are dependent upon two observations. Their use of a biodegradable nanofibrous scaffold contributed to the increasing content of proteoglycan and collagen II over the culture period of 70 days. Furthermore, some of the donors came from knee arthroplasty patients, and thus there were resections with large amounts of meniscus substance. Nevertheless, there was significant data variation relating to these observations and the continual passaging of meniscal cells increases the risk of cell dedifferentiation to obtain sufficient cell numbers for further culture and analysis.

In the present study, an in-vitro evaluation of human meniscal tissue from non-refixable tears was used to assess their potential in a clinical setting. However, the human meniscal cell pellets cultured in chondrogenic medium revealed moderate gene expression and no deposition of collagen II after 21 days (Figs. 4, 5 and 6). As previously discussed, monolayer expansion to achieve sufficient cell numbers for aggregate formation increases dedifferentiation and may have led to poor outcomes in vitro despite the presence of TGF-β. These results are also contrary to our in-vivo data or similar studies using meniscus tissue, and thus indicate that this may not be an appropriate cell source for meniscus repair in a clinical setting.

An alternative cell source for meniscus repair are MSCs, and these have been shown to induce meniscus regeneration [11–13, 28]. MSCs have been detected in vivo following meniscal lesions in the knee synovial fluid [21], whilst a progenitor population has been identified within the avascular region of the injured meniscus that had high migratory potential towards the lesion [38]. However, our study focused on the MSCs derived from the bone marrow, which show the potential to differentiate into bone, adipose tissue, and cartilage in this setting [13]. These cells demonstrated meniscus-like repair in the punch defect after 3 months in vivo. This confirms the results of previously published studies on different meniscus defect types that all showed that untreated injuries showed no healing, with a “non-union” of the lesion comparable to the human situation [11–13, 28]. Furthermore, in these studies, the application of a MSC-based treatment revealed significantly superior results compared to the use of a cell-free scaffold. Therefore, the described animal model can be considered as appropriate for the evaluation of the regenerative potential of different meniscus treatment options. As the historical controls with untreated defects and treatment with cell-free scaffolds showed only a reduced quality of regeneration with nonintegrated fibrous tissue, different cell-based repair strategies should be tested in this study. Meniscal cells and MSCs differentiated and integrated meniscus-like repair tissue, with scoring results between 12 and 16 points after 3 months. In comparison, the historical controls of cell-free treatment showed scoring results of only 8 points, indicating an improvement in meniscus regeneration using a cell-based repair strategy. In addition, the demanding early osteoarthritis situation within the model provides further evidence of the suitability of MSCs within a clinical context.

Previous studies have reported the positive effects of MSCs on meniscus regeneration both in vitro and in vivo and from different sources, including adipose and synovium [18, 39, 40]. Gonzalez-Fernandez et al. compared the regeneration potential induced by bone marrow-derived MSCs to adipose tissue-derived stem cells in an equine model and found no differences between the two cell sources [19]. As previously stated, the feasibility within a degenerative situation was not assessed in their equine model. The reasons for the successful repair of the meniscus using MSCs may be related to two distinct mechanisms. MSCs may have differentiated into meniscal cells due to the surrounding tissue matrix, and cell-cell communication or the secretion of trophic factors released by MSCs may have helped to heal the meniscus via pharmacological means or via recruitment of resident cell populations [41].

As MSCs from many sources show positive results regarding the enhancement of meniscal repair it seems that the question is not the origin of the progenitor cells but more their availability and applicability for clinical use. In other connective tissues, such as articular cartilage, preliminary clinical data show positive effects on regeneration with the application of MSCs. Sekiya et al. detected significant improvements after arthroscopic transplantation of synovial-derived stem cells in small (average 200 mm^2^) cartilage defects by magnetic resonance imaging (MRI) scoring, qualitative histology, and Lysholm score evaluation [42]. A further advantage of autologous MSCs is the potential for a single-step cell-based repair augmentation since they have a higher proliferation rate than meniscal cells and are less susceptible to dedifferentiation [43]. Our in-vitro results confirm the high chondrogenic potential of human MSCs and their qualification for augmentation of meniscal healing in a clinical setting.

However, limited data are available on the clinical use of MSCs for meniscus regeneration. Whitehouse et al. [44] conducted an open-label first-in-human study for repair of avascular meniscal lesions with autologous MSCs. Following isolation and expansion, MSCs from iliac crest bone marrow were seeded on collagen scaffolds. These cell-matrix constructs were placed and sutured into avascular meniscal tears of five patients. After 2 years, three patients were asymptomatic with no signs of a recurrent tear in the MRI control compared to two patients that required subsequent meniscectomy due to re-tear or nonhealing. In a controlled randomized trial, Vangsness et al. delivered allogenic MSCs 1 week after arthroscopic partial medial meniscectomy via intra-articular injection to the knee. A year after surgery, a significantly increased meniscal volume determined by quantitative MRI was detected in 24% of patients in the treatment group, while no patient in the control group showed an increasing amount of meniscus tissue. Additionally, stem cell injection revealed beneficial effects on pain management of these patients after partial meniscectomy [45].

A limitation of this study is the animal model and the comparison to a historical control. However, the study shows that regeneration of avascular meniscal defects is possible by a cell-based treatment even in a demanding and clinically very relevant situation such as early osteoarthritis. A strength of the study is the analysis of different human cell sources that may be used for an autologous cell-based meniscus treatment using meniscus cells or MSCs. In comparison to MSCs, human meniscal cells from non-refixable meniscal tears or meniscal debris demonstrated a very limited capacity for chondrogenic differentiation. Thus, MSCs appear to be the most promising cell source for an autologous cell-based meniscus treatment approach, including low donor site morbidity. However, there are disadvantages that limit their clinical applicability, particularly the high treatment costs, requirement for cell expansion prior to application, and regulatory burdens that currently inhibit their use in daily clinical practice [43]. These problems should be resolved to facilitate the cell-based treatment of meniscal defects with MSCs and therefore improve the clinical outcome of this common injury.

## Conclusions

Cell-based treatment of meniscal defects with autologous meniscal cells and MSCs showed equally positive effects on meniscus regeneration in an animal model in a situation of early osteoarthritis. The defects were completely filled with differentiated meniscus-like tissue in both groups. However, meniscal cell harvest revealed inacceptable donor side morbidity causing the early osteoarthritic changes. Additionally, human meniscal cells derived from nonrepairable meniscal tears showed no chondrogenic potential using ELISA and real-time PCR analysis. In contrast, due to their high regenerative capability and promotion of meniscal healing, MSCs appear to be a more appropriate source for cell-based treatment of the meniscus in a clinical setting of early osteoarthritis.

## Abbreviations

DMEM: Dulbecco’s modified Eagle’s medium
DMMB: 1,9-Dimethylmethylene blue
ELISA: Enzyme-linked immunosorbent assay
FBS: Fetal bovine serum
MRI: Magnetic resonance imaging
MSC: Mesenchymal stem cell
PBS: Phosphate-buffered saline
PCR: Polymerase chain reaction
sGAG: Sulfated glycosaminoglycans
TGF: Transforming growth factor
